# Identifying vital nodes for yeast network by dynamic network entropy

**DOI:** 10.1186/s12859-024-05863-x

**Published:** 2024-07-18

**Authors:** Jingchen Liu, Yan Wang, Jiali Men, Haohua Wang

**Affiliations:** 1 School of Mathematics and Statistics, Hainan University, Haikou, 570228 Hainan People’s Republic of China; 2 Department of Neurology, The First Affiliated Hospital, University of South China, Hengyang, 421001 Hunan People’s Republic of China; 3 School of Life Sciences, Hainan University, Haikou, 570228 Hainan People’s Republic of China; 4 Key Laboratory of Engineering Modeling and Statistical Computation of Hainan Province, Hainan University, Haikou, 570228 Hainan People’s Republic of China; 5 School of Mathematics, Shandong University, Jinan, 250100 Shandong People’s Republic of China

**Keywords:** Network entropy, Gene regulatory network, K2 algorithm, Partial least squares, Network simulation, Time series plateau interval

## Abstract

**Background:**

The progress of the cell cycle of yeast involves the regulatory relationships between genes and the interactions proteins. However, it is still obscure which type of protein plays a decisive role in regulation and how to identify the vital nodes in the regulatory network. To elucidate the sensitive node or gene in the progression of yeast, here, we select 8 crucial regulatory factors from the yeast cell cycle to decipher a specific network and propose a simple mixed K2 algorithm to identify effectively the sensitive nodes and genes in the evolution of yeast.

**Results:**

Considering the multivariate of cell cycle data, we first utilize the K2 algorithm limited to the stationary interval for the time series segmentation to measure the scores for refining the specific network. After that, we employ the network entropy to effectively screen the obtained specific network, and simulate the gene expression data by a normal distribution approximation and the screened specific network by the partial least squares method. We can conclude that the robustness of the specific network screened by network entropy is better than that of the specific network with the determined relationship by comparing the obtained specific network with the determined relationship. Finally, we can determine that the node CDH1 has the highest score in the specific network through a sensitivity score calculated by network entropy implying the gene CDH1 is the most sensitive regulatory factor.

**Conclusions:**

It is clearly of great potential value to reconstruct and visualize gene regulatory networks according to gene databases for life activities. Here, we present an available algorithm to achieve the network reconstruction by measuring the network entropy and identifying the vital nodes in the specific nodes. The results indicate that inhibiting or enhancing the expression of CDH1 can maximize the inhibition or enhancement of the yeast cell cycle. Although our algorithm is simple, it is also the first step in deciphering the profound mystery of gene regulation.

## Background

After entering the post-genomic era, the main research content of bioinformatics is to analyze a large amount of various biological molecular data and deeply explore the life information contained in it. It is a relatively new and popular research issue to put forward some efficient arithmetic and reconstruct the gene regulatory network [1]. It is known that the specific network in the gene regulatory network can always perform some specific functions, such as the cell cycle, biological clock, etc. [2, 3]. In an organism, the expression regulation of any gene is not isolated but is inevitably promoted or suppressed by other genes [1–3]. Therefore, it is the first goal in this field to reverse-mine the association between genes within biological cells based on existing known gene expression data to scaffold the specific network and determine the sensitive nodes or biomarker protein, and then visualize the interactions between genes in the form of network graphs to reveal the functional information of various genes in biological cells in life activities.

In recent years, it has gradually advanced of that the relevant research methods for gene regulatory networks. Several mathematical models have been applied to target gene regulatory network modeling, which is famous for Boolean network models [2, 3], neural network models [4], differential equation models (based on ordinary differential equations (ODEs)) [5], and probabilistic graphical models [6]. These models achieve partly the abstraction of the real gene regulatory network to different degrees. Among them, the Bayesian network model has been widely used and is a mainstream method to study gene regulation because of its characteristic of high scaffolding efficiency and high accuracy of results compared with other models [7].

In 1992, Cooper et al. [8] proposed the K2 algorithm to learn the Bayesian network structure for building a specific network. Although the K2 algorithm is famous for its high execution efficiency, it does not take into account the characteristics of the time-variant, that is, the structure of the corresponding regulatory network can change with time which results in the problem of learning excessively easily. Wang proposed an AutoDBN algorithm to learn dynamic Bayesian networks with variable structures [9]. The AutoDBN algorithm introduces manifold theory to partition the stationarity of time series [10]. Although this method investigates the stationarity partition of time series, it does not discuss the changes of models over time on adjacent stationary time periods. Lau et al. [11] introduced entropy into the scaffolding of the gene regulatory network, however, it is easy to lead to error results for this method because they only employ the Boolean network to define the internal genetic function and interactions as simple logical rules that can be inferred from the gene expression level of each gene determining one logical rule.

With the development of the next sequencing technology (NGS), the gene sequencing data have the properties of multivariate and non-homogeneous and it is a pressing matter of the moment to develop an algorithm to compensate for the drawback of the scaffolding efficiency and discriminate the important nodes in a specific network. Without loss of generality, we select the classical gene module of yeast to exhibit the robustness and efficiency of our algorithm. The reason why we chose the yeast module is that it is of great significance for human production and life, and it has the characteristics of a small genome and easy cultivation, as well as also directly the cell cycle progress [12]. In fact, the specific network regulates the entire cell cycle: during the G1 phase, CDC28 gene expression is transcribed and forms a complex with CLN3. When the level of the complex exceeds a certain threshold, it phosphorylates SBF [11] and MBF [13] to trigger the G1 to S transition. Subsequently, SBF and MBF promote the transcription of CLN1 and CLN2 [14]. At the same time, the synergistic effect of CDH1 and APC controls the degradation of M G1-related proteins [15, 16]. Then, CLN1, CLN2, and CDC28 interact to form a complex to promote the activation of CDK, thus driving DNA replication and entering mitosis [17]. In the G2 phase, the rise of CDC28-related compounds led to the inactivation of SBF, and then the activity of CLN1 and CLN2 decreased [18]. Subsequently, CDH1 undergoes phosphorylation and is subsequently inactivated by CLN1 and CLN2 [19, 20]. In the G2 phase stage, the membrane filament assembly defect of the bud neck led to the low phosphorylation and stability of SWE1, leading to the dependent inhibition of CLB-CDC28 by SWE1. CDC5-related genes were expressed and reached a certain number in the G2 phase, then Cdc5-mediated phosphorylation promoted the down-regulation of SWE1, promoted the effective degradation of Swe1, and effectively activated CLB-CDC28 [21, 22]. Then, the yeast enters the M phase. SWI5 is the SIC1 transcription factor. Once SWI5 enters the cell, it will be destroyed. SWI5 will promote the cell to return from the M phase to the G1 phase [22]. In addition, CDH1 and SIC1 cooperate to promote origin redundancy in the cell cycle to prevent a shortage of active origin regions and maintain chromosome stability [23].

**Fig. 1:**
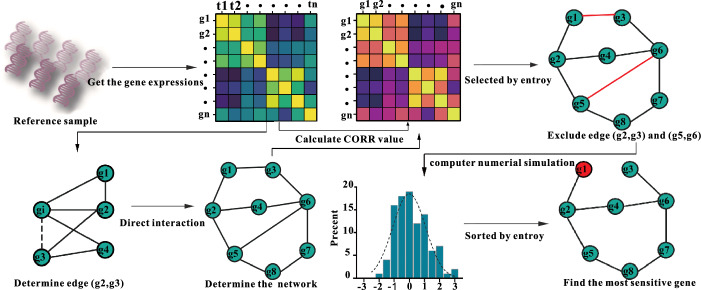
Flow diagram for scaffolding and selecting the specific network. The time-dependent expression data of a set of genes are acquired, which is built after the data processing using the K2 algorithm based on the time series plateau interval. The obtained network was selected using network entropy. Numerical simulations were subsequently performed using a normal distribution with partial least squares. Finally, gene sensitivities were ranked using network entropy

Here, we integrate the existing algorithms to build up the network by introducing the time series plateau interval into the K2 algorithm and then screen the built regulatory network by network entropy to try to surmount the problems of the overlearning problem and low construction efficiency of P-BIC scores, as well as the problem of the Boolean network logic rules to infer error conclusion (referring to Fig. 1). We select eight specific networks of genes: CLN1, CLN2, CDC28, SWE1, CDC5, CDH1, SWI5, and SIC1 (refer to Abbreviations), to investigate the gene regulation of the yeast cell cycle. Also, we calculate the network entropy to screen the stability of a specific network and then simulate the progress of yeast by normal distribution approximation to determine the specific network. By comparing in pairs the networks, we can yield the sorting of gene sensitivity and identify the vital nodes or genes in the specific network. For the yeast cell cycle network, we can find that node CDH1 is the first sensitive gene that can achieve effectively the maximization of the inhibition or enhancement of the yeast cell cycle.

## Materials and methods

### K2 algorithm

We employ the K2 algorithm to construct the specific network [8]. The K2 algorithm discusses a defined scoring function, starting from an empty specific network, and selects the upstream gene of the given gene that maximizes the posterior structure probability based on the order of upstream and downstream genes. Traverse all genes in sequence, gradually adding the best upstream gene for each gene [8, 24].

The original scoring function is $score(i,\pi_{i} ) = \Pi_{j = 1}^{{q_{i} }} \frac{{(r_{i} - 1)!}}{{(N_{ij} + r_{i} - 1)!}}\Pi_{k = 1}^{{r_{j} }} N_{ijk} !$ where $N_{ij} = \mathop \sum \limits_{k = 1}^{{r_{1} }} N_{ijk}$, $i$ representing gene $i$, $\pi_{i}$ representing the upstream genes of the gene $i$, $n$ representing the number of genes, $q_{i}$ representing the number of types of upstream genes of the gene $i$, $r_{i}$ is the state of the gene $i$ where the state is expressed and unexpressed, i.e. $r_{i}$ is taken as 2 or 3.  $N_{ijk}$ representing the kth state of the gene.

### Segmentation of multivariate time series

Time series stationarity is a concept in time series analysis [25]. Time series stationarity was introduced into gene expression levels [9, 26]. The ratio of the magnitude of fluctuation of a gene's expression level over a given period to that of the entire period may reflect a plateau in this gene's expression. The smaller ratios indicate a plateau in this gene's expression level over a selected period, which is referred to as the plateau in this gene's expression [25, 26]. Within the interval [*s*, *k*] and [*k*, *t*], the stationarity $RB(s,t)$ and $RB(k,t)$ are calculated, respectively. The expression levels of all genes are stationary within [*s*, *t*] if $RB(s,k)$ is the same distribution function as $RB(k,t)$. That is, [*s*, *k*] and [*k*, *t*] are within a stationary interval and the two intervals can be merged into a single interval [*s*, *t*]. Here, the stationarity is defined as$RB(s,k) = \frac{{(\mathop \sum \limits_{i \in [s,t]}^{{}} |G(i) - \mu_{G} (s,t)|)/n}}{{(\mathop \sum \limits_{i \in [1,t]}^{{}} |G(i) - \mu_{G} (1,T)|)/N}}$, where $\mu_{G} (s,t) = \frac{1}{n}\mathop \sum \limits_{i \in [s,t]}^{{}} G(i)$, $G(i)$representing the expression of gen $G$ at the point $i$. The value $RB(s,k)$ is smaller, the more stable the level of change in the gene $i$ within [*s*, *t*]. The value $RB(s,k)$ is larger, indicating that the level of change in gene $i$fluctuates more within [*s*, *t*] [9, 26].

### Evaluation by network entropy

The concept of entropy stems from thermodynamics, measuring the degree of energy failure in a system of matter [27]. It is essentially a system’s “degree of intrinsic disorder”. Entropy is introduced into networks to solve various problems [28]. It is known from the network entropy definition that the network entropy energy can describe the stability of a specific network, i.e., the smaller the network entropy, the stronger the stability of that network. For a specific network, the network entropy is calculated as:

1 $$H_{i} = - \sum\limits_{j = 1}^{N} {p_{ij} \log (p_{ij} )}$$

2 $${\text{with}}\,p_{ij} = \frac{{{|}CORR(k_{i} ,k_{j} ){|}}}{{\sum\limits_{m = 1}^{N} {{|}CORR(k_{i} ,k_{j} ){|}} }}$$

where $$k_{i}$$represents the expression of gene $$i$$ changes over time data, and if the gene $$i$$is connected with the gene $$j$$ without an edge, the calculation is not performed. Otherwise, if data $$k_{i}$$$$k_{j}$$ are all normally distributed, $$CORR(k_{i} ,k_{j} )$$ represents the Pearson correlation coefficient of the amount of expression between gene $$i$$ and gene $$j$$. Or else, if data $$k_{i}$$$$k_{j}$$ are not normally distributed, $$CORR(k_{i} ,k_{j} )$$ represents the Spearman correlation coefficient of the amount of expression between the gene $$i$$ and the gene $$j$$. $$N$$ represents the number of selected genes, and $$H_{i}$$ represents the network entropy of the gene $$i$$.

### Algorithms for specific network selecting

Given an initial specific network with the dynamic change of gene expression quantity, we screen the network in the following steps:

1. Set *n* = number of unconfirmed edges, threshold *a* and *b*, number of the unconfirmed edges;
2. Create a network NET composed of correct edges and a zero matrix Initial of *a* × *n*;
3. Do the following for the unconfirmed edges to traverse through all unconfirmed edges:
  3.1 setting *m* = 1;
  3.2 Perform the following for the selected unconfirmed edges, to ensure all unconfirmed edge combinations are considered in full:
    3.2.1 NET1 = NET adds m unconfirmed edges except for this unconfirmed edge;
    3.2.2 NET2 = NET1 added this unconfirmed edge;
    3.2.3 Setting *c*, *d* = NET1 network entropy, NET2 network entropy;
    3.2.4 If *c*-*d* > *a*, Initial (*i*) = 0; Otherwise Initial (*i*) = Initial (*i*). Turn step 3.2;
  3.3 If *m* < *n*-1, then *n* = *m* + 1, turn step 3.2; Otherwise turn step 3.4;
  3.4 If the presence of an unconfirmed edge is not selected, select the next unconfirmed edge, step 3; Otherwise, turn to step 4;
4. If Initial (i) < b, join the edge to the network net; Otherwise, do not operate on NET;
5. Output network NET.

### Specific network-sensitive gene ranking algorithm

For the dynamic changes in the amount of gene expression of screened networks, we use the following algorithm to rank the level of sensitivity of the gene model:

1. Create a network NET composed of the selected rear edges, *n* = the number of genes of that network;
2. *c* = network entropy of NET, *i* = 1, genes are numbered to create a 1 × n zero vector *k*;
3. Do the following for nodes of a network net, to all nodes be traversed:
  3.1 Selection of the Gene i;
  3.2 NET1 = Remove the node and corresponding out edge, into the edge;
  3.3 *d* = network NET1 network entropy;
  3.4 g = *c*–*d*;
  3.5 If *i* < *n*, *i* = *i* + 1, turn step 3; Otherwise turn step 4;
4. Output after sorting vector k elements.

### Data source and processing

**Fig. 2:**
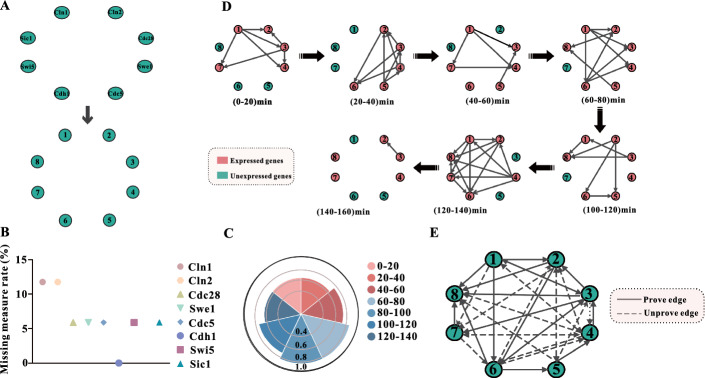
**A** The eight genes were numbered, and CLN1, CLN2, CDC28, SWE1, CDC5, CDH1, SWI5, and SIC1 were assigned values of 1, 2, 3, 4, 5, 6, 7, 8. **B** The missing data rate of the eight genes. **C** The proportion of expressed genes in each interval after time series stationary interval segmentation. **D** Scaffolding process of the specific network by K2 algorithm based on the time series stationary interval. Red indicates expressed genes and green indicates non-expressed genes. **E** Synthesis of the resulting network. The solid line represents the proven relationship and the dotted line represents the unconfirmed relationship

The species data applied here are microarray gene expression data for yeast cells derived from Spellman et al.’s experiments [29]. This dataset is expression data for a total of 6178 genes resulting from 6 different conditions. Here, we select eight genes from the CDC28 dataset [28]: CLN1, CLN2, CDC28, SWE1, CDC5, CDH1, SWI5, and SIC1 and numbered the genes (Fig. 2A).

First, the selected genes are filtered, and the missing data rate is lower than 15% in all eight genes (Fig. 2B), which indicates that the selected genes all satisfy the conditions. A cubic linear function is subsequently utilized to impute missing data for the CDC28 dataset and to normalize the post-imputed data. Finally, the data are discretized, we choose the three-value discretization method to discretize the data:

$$\left\{ {\begin{array}{*{20}l} {a_{ij} = 3} \hfill & {a_{ij} > \mu_{i} + s_{i} } \hfill \\ \begin{gathered} a_{ij} = 2 \hfill \\ a_{ij} = 1 \hfill \\ \end{gathered} \hfill & \begin{gathered} a_{ij} = \mu_{i} + s_{i} \hfill \\ a_{ij} < \mu_{i} + s_{i} \hfill \\ \end{gathered} \hfill \\ \end{array} } \right.$$

where $a_{ij}$is the value of the gene $i$ at the time $j$, $\mu_{i}$ is the mean of the gene $i$ expression abundance over time, and $s_{i}$ is the variance of the gene $i$ expression abundance over time.

## Result

### Specific network time division based on time series plateau interval

The selected 8 genes are subjected to segmentation of the time series plateau interval. A *p* value of 0.01 is set to segment the time-series data set into 7 plateaus. Since [60, 80] is merged into a plateau interval sheet with [80, 100], we can consider gene expression at [60, 80] as that at [60, 100]. Therefore, we divide the time into [0, 20], [20, 40], [40, 60], [60, 100], [100, 120], [120, 140], [140, 160] seven time periods. The activity rates of the genes stabilized between 60 and 90% across the respective plateau intervals(Fig. 2C), implying that the partitioned plateau sheets all satisfied the requirements. The expressed genes in the seven-time periods are shown in red in Fig. 2D. Since it is not clear the stages of the cell cycle at the beginning of the experiment, the cell cycle periods for the respective periods could not be determined. [0, 20], [20, 40], and [40, 60], in these three intervals, the active genes are mainly expressed as three genes CLN1, CLN2, and CDC28, which are speculated to be probably from the G1 phase to S phase [30]. [60, 100] and [100, 120], the two individual interval regulatory processes are complex, and almost all genes are involved in the expression. Within these two intervals, it is known by CDC5 gene expression that the stage is in the S phase and G2 phase [22]. Also by the fact that the SWI5 gene is not expressed in these two intervals, it was judged that this interval may be the S phase versus the early middle G2 phase [30]. During the interval [120, 140] and [140, 160], CDH1, SWI5, and SIC1 are more strongly expressed, inferring the G2 phase, M phase, and the early G1 phase [31].

### K2 algorithm to construct the specificity network

Based on the segmentation results of the time series plateau interval of the gene expression data of CLN1, CLN2, CDC28, SWE1, CDC5, CDH1, SWI5, and SIC1, relevant computer programs are written using the BNT toolbox in MATLAB. This specific network over time is shown in Fig. 2D.

It is clear that 0–60 min is predominantly the mid-late G1 phase. This process mainly involves the activation of cyclin CDC28 kinase by CLN1 and CLN2 kinases and the accumulation of CDC28-associated proteins. When the CDC28 protein passes a certain threshold, the related genes that it regulates become activated to promote the transcription of CLN1, CLN2, and other genes required for S phase progression. At the same time, CLN1 and CLN2 interact with CDC28 to promote the activation of B-type cyclin-associated CDKs, which bind to CDC28 expressing proteins and promote the transition of the cell cycle from the G1 phase to the S phase [30, 31]. SWE1 is also expressed starting in the late G1 phase [22].

Followed by 60–120 min, it is predominantly in the S phase with early G2 phase. Multiple genes are expressed continuously during this period. During the S phase, SWE1-related genes continue to be expressed and accumulate, become sequentially hyperphosphorylated, give rise to multiple isoforms, and then undergo ubiquitin-mediated degradation. Defective septal filament assembly at the bud neck leads to hypophosphorylation and stabilization of SWE1 and, as a result, SWE1-dependent inhibition of CLB-CDC28. In parallel, CDC5-associated genes are expressed and reach a certain number in the G2 phase, and subsequent CDC5-mediated phosphorylation prompts SWE1 downregulation, promoting efficient degradation of SWE1 for efficient activation by CLB-CDC28 [21, 22].

From 120 to 160 min is mainly in the mid-late G2 phase, M phase to early G1 phase. SWI5 begins to be expressed during the G2 phase, and the mRNA level of SWI5 peaks in G2/M, with nascent proteins entering the nucleus and promoting the transcription of SIC1 and many other periodically expressed genes. This results in an M/G1 specific transcriptional burst of SIC1, which encodes a potent B-type cell cycle kinase inhibitor. SWI5, SIC1, and CDH1 subsequently dephosphorylate, leading to the inhibition of CDC28 and degradation of cyclins required for mitotic exit. SIC1 and APC activities persist through G1, resulting in a B-type cell cycle kinase deficient state required for the establishment of the pre-replication complex on genomic DNA [30]. It can be known that the constructing network process coincides with the cell cycle, which proves the correctness of our used method to some extent.

**Fig. 3:**
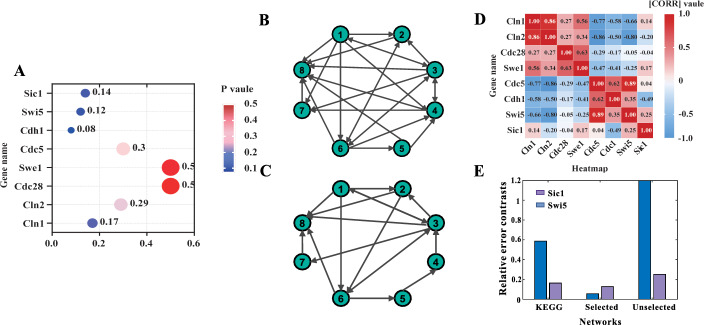
**A** Results were tested for normal distribution of gene expression. **B** Specificity network after selecting by network entropy. **C** Network of KEGG, EVEX, and relevant literature. **D** Heat map of correlation coefficients for expression quantities between individual genes. **E** Simulated network relative error contrasts

The resulting network is shown in Fig. 2E. From the experimental results (Fig. 2E), we can yield that the network constructed by the improved K2 algorithm has a total of 34 regulatory relationships. We use the protein interaction relationships of KEGG and corresponding literature as prior information and fuse the results of EVEX data mining to obtain a deterministic relationship network (Fig. 3C) [32, 33]. Comparing the experiments with known networks inferred from the literature indicates that 17 regulatory relationships have been proven in biological experiments, but there are still 17 relationships that have not been proven, with an accuracy rate of 50%. The results are compared with the REVEAL algorithm 36% correct [33] and the DBCMC algorithm 29% correct [34], and the method presented here has a higher correct rate than the REVEAL algorithm and the DBCMC algorithm, implying the method presented here is effective.

### Selecting of networks using network entropy

First, the level of gene expression is tested for *L* normal distribution. We selected all the data with *p* values greater than 0.05 at the 95% confidence level from the normal distribution test results (Fig. 3A), that is, the selected data are all normally distributed. We subsequently calculate the entropy values of the respective genes using Eq. (1) and select this specific network following the algorithm of network screening for specificity. Considering the number of unproven edges in the network, we divide it into two groups. The first one includes eight uncertain edges that set the threshold for *a* at 0.3 and threshold *b* at 100. The other nine edges are the second group with a threshold of *a* at 0.3 and a threshold of *b* at 200. The resulting specific network after selection is shown in Fig. 3 B. From the experimental results, after the network entropy selection, eight relationships are added: CDH1 regulates CLN2, CLN1 regulates SWE1, CDC28 regulates SWE1, CDH1 regulates SWE1, CLN1 regulate SWI5, SWE1 regulate SWI5, CDC5 regulate SIC1, SWE1 regulate SIC1. Among them, Skotheim et al. [35] demonstrated that CDH1 mutations can partially salvage G2 stagnation in CLN1/CLN2 dual mutants, indicating that CDH1 regulation of CLN2 may exist. Ahn et al. [36] demonstrated that when using wild-type CDC28, CLN1 overexpression-induced silk formation is significantly reduced in SWE1 deficiency, meaning a certain regulatory relationship among CDC28, SWE1, and CLN. The other sets of relationships have not been experimentally proven, so a definitive relationship cannot be obtained. The above results indicate that the network selected by our method is correct in biological significance, which is helpful for the relationship between gene regulation.

### Numeric simulation by partial least squares (PLS) for selecting a specific network

To verify that the selected specific network is mathematically correct, the resulting network is simulated by partial least squares (PLS). PLS integrates the expression data between the gene and other genes to establish a linear equation:

$$x_{i} (t) = \beta_{1} x_{1} (t) + \beta_{2} x_{2} (t) + \ldots + \beta_{i - 1} x_{i - 1} (t) + \beta_{i + 1} x_{i + 1} (t) + \ldots + \beta_{n} x_{n} (t)$$

where the $x_{i} (t)$ represents the expression level of the gene $i$ at the time $t$; the $\beta_{j}$ represents the coefficient and takes the value of 0 if the gene $j$ is not upstream of the gene $i$; $n$ represents the total number of genes of the specific network.

Since the gene expression level conforms to the normal distribution, we use the normal distribution to generate a set of data and then put the data into the established PLS model to compare the average relative error of genes and obtain the network error. Comparing the relative error obtained with the standard network (Fig. 2C), we can see the network relative error of the entropy screening of the known network is better than the other two errors (Fig. 2D), implying that the robustness of the screened network is higher than the other two networks.

### Node sensitivity ranking based on network entropy

**Fig. 4:**
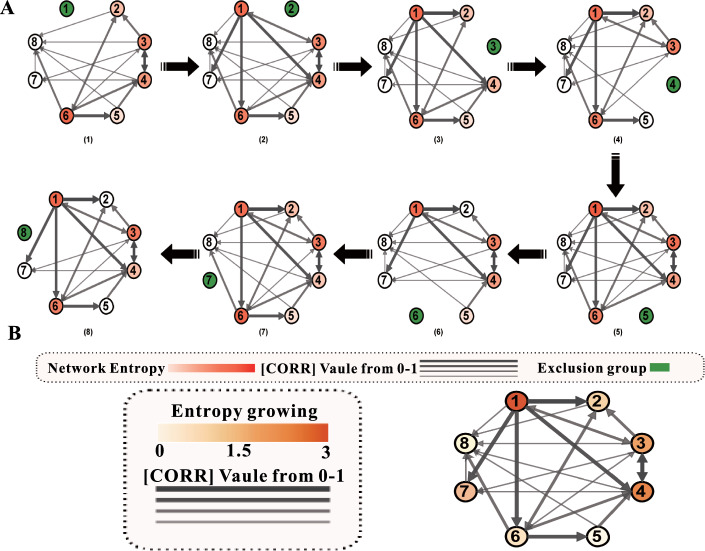
**A** Network evolution of specificity based on network entropy. **B** Results of gene sensitivity ranking based on network entropy

For the screened network, we used network entropy to rank their degree of gene sensitivity. The greater the junction network entropy, the worse its stability. We have to remove the junction that reduces the largest in-network entropy (namely, the node sensitivity is greater), i.e., the greater the increase in network entropy upon inclusion of this gene, the more sensitive it is. We sort the network genes by a specific network sensitive-gene ranking algorithm. The genes are sequentially deleted in specific network evolution based on network entropy, as shown in Fig. 4A. We start this procedure from gene 1, and the rest of the genes are retained. Then, we calculate their entropy cyclically, the sequencing results are shown in Fig. 4B. From the above experiments, the sensitivity of genes sorts from small to large as CDC5, SWI5, SIC1, CDC5, CLN1, CDCD28, and CLN1.

It is known that if we are to inhibit the activity of this network, then we should preferentially repress the gene CDH1, thus minimizing the entropy of this specific network. CDH1 promotes APC/C production in the late stage of mitosis and serves as an antagonist to the checkpoint of spindle components, guiding the ubiquitination of cell cycle proteins, and resulting in mitotic exit. It targets specific substrates including CDC20p, ASE1p, CIN8p, FIN1p, and CLB5p [15, 37–39]. CDH1 plays a crucial role throughout the entire cell cycle, which verifies our results to some extent.

## Conclusions

Gene regulatory relationships, as a means of mining living information, have been a research hotspot for the past few years. It provides important support and reference for the study of gene regulatory relationships to find sensitive nodes or genes in specific networks. However, because the methods of gene chip data and specific network construction are affected by many factors, it is often difficult for the traditional model to build a correct specific network and search for sensitive genes. In addition, traditional algorithms do not take into account the problems that the regulatory relationship between genes often changes over time, and the structure of the corresponding regulatory network also changes over time. So for the specific network scaffolding in yeast, here, we propose a mixed K2 algorithm based on the time-series stationary interval segmentation to screen the specific network after scaffolding using the network entropy. To further find the sensitive genes of this yeast-specific network, we sort the specific network-sensitive genes by the method of network entropy. The results indicate that the mixed K2 algorithm can solve effectively the problem overlearning problem and low scaffolding efficiency, as well as the problem of the Boolean network single logic rule. Furthermore, we calculate the value of network entropy to measure the stability of the specific network obtained by adding or deleting the edges of a determined network. We notice that the more sensitive the node is, the greater the reduction of network entropy after removing a certain gene of this specific network. Like this, we can identify the most sensitive gene of this specific network as CDH1, and to some extent, it has been proved by related literature [15, 16]. Lastly, we also validate the results through simulation by the partial least squares and the accuracy is higher than the existing results.

## Discussion

It is of great potential value for humans to study gene regulatory networks. Undoubtedly, it can help humans reconstruct and visualize gene regulatory networks using gene databases and further understand the complex regulatory relationships among various types of life activities of cells at the gene level, such as the deep regulation of DNA transcription and mRNA translation [40–43]. Also, it can help to understand the complex disease from the gene level with the help of a directed acyclic graph structure abstracted from gene regulatory networks, including the generation of tumors in cancer and the differentiation of cancer cells, as well as helping humans to target it for therapy [44, 45]. A relatively mature network is a death signaling network that contains the relation between RIP1 level and the occurrence of necroptosis to reveal biphasic cell apoptosis and necrosis pathways [44]. What is more, it is helpful to strengthen the pertinence of drug design with the help of this tool of gene regulatory networks and designing the corresponding target screening algorithms, to develop drugs at a smaller cost, and improve the efficiency of drug research and development [46]. The key problem in drug design is to identify the most sensitive biomarker gene in the corresponding regulatory network. For example, curcumin, as a potentially promising anticancer drug, is from 5450 natural small molecules. There is a key biomarker target BIRC5 (survivin) for curcumin that is selected from the human transcriptional regulatory network (HTRN) by the random walk-based graph embedding method to calculate the diffusion profiles of drugs and cancers [47].

Moreover, the construction of a yeast-specific network and the evaluation of sensitive genes depend partly on the threshold parameters* a* and *b*, which are too large to fail to select the existing relationship of genes and too small to delete the redundant edge in the specific network, ensuring that the algorithm designed here can be applied to small-scale data without causing overfitting [48–50]. Therefore, how to set reasonable parameters is the key to applying the related algorithm and we can focus on setting parameters *a* and *b* in future experiments to improve the screened specific network accuracy. Furthermore, it is feasible from the viewpoint of mathematics and a certain biological sense to rank the sensitivity of genes by network entropy. We need to validate further in the sense of vivo biological experiments by comparing our results with gene deletion that represses gene expression the most. It is worth noting that the mixed K2 algorithm gives a canonical form to identify the vital nodes in individual regulatory factors, however, the gene regulation is often multiplexed and cell fate is determined by recombination of regulatory factors [51–54]. We will combine our algorithm with SWATH-MS technology in the next step to apply it to large-scale networks to further investigate changes in cell life states through transitions between cell states, and identify key nodes in the process of cell state transition [52–56].

## Data Availability

The datasets supporting the conclusions of this article are included with the article. Project name: DNE. Project home page: https://github.com/huazi8112/DNE. Project inclusion: All datasets and the code needed to replicate the experiment.

## Abbreviations

APC/C: Anaphase-promoting complex/cyclosome
ASE1: Anaphase spindle elongation 1
CLN1: Cyclin 1
CLN2: Cyclin 2
CLN3: Cyclin 3
CLB: Cyclin B
CIN8: Kinesin motor protein CIN8
CDC28: Cyclin-dependent serine/threonine-protein kinase CDC28
CDC5: Polo kinase CDC5
CDH1: CDC20 homology 1
DNA: Deoxyribonucleic acid
mRNA: Messenger RNA
MBF: MCB-binding factor
FIN1: Filaments in between nuclei 1
SBF: SCB-binding factor
SIC1: Substrate/subunit inhibitor of cyclin-dependent protein kinase 1
SWI5: Regulatory protein SWI5
SWE1: Saccharomyces Wee1
PLS: Partial least squares
